# Status Dystonicus as an Acute Sequelae Following Anoxic Cerebral Damage

**DOI:** 10.5334/tohm.564

**Published:** 2019-04-15

**Authors:** Somdattaa Ray, Ravinder Jeet Singh Sidhu, Pramod Kumar Pal, Ravi Yadav

**Affiliations:** 1 Department of Neurology, National Institute of Mental Health and Neurosciences (NIMHANS), Bangalore, IN

**Keywords:** Dystonia, emergency, hypoxia, status, anoxia

## Abstract

**Background:**

Status dystonicus (SD) is the term used for extreme, continuous, generalized muscle contractions that are poorly responsive to treatment. Here, we report a rare case of acute hypoxic ischemic encephalopathy presenting with SD.

**Case Report:**

A young male sustained cerebral hypoxia following a cardiac event and presented with opisthotonic posturing and dystonia refractory to medical therapy. His serum creatine phosphokinase was high and his urine tested positive for myoglobin.

**Discussion:**

SD as an acute sequelae following acute brain hypoxia is rare. Management of brain anoxia is challenging, even more so when the presentation is compounded by refractory SD.

## Introduction

Continuous or intermittent muscle contractions resulting in abnormal and repeated patterened movements characterize dystonia that can progressively worsen causing extreme, generalized continuous spasms termed status dystonicus (SD).^1^,^2^,^3^,^24^ SD is a potentially life- threatening crisis. Rhabdomyolysis, myoglobinuria, and hyperpyrexia are a few additional features that *may* be associated with SD.^4^,^5^ Secondary dystonia is the most common underlying cause of SD, with cerebral palsy the cause in 59.3% of patients.^6^

Vascular and metabolic theories have been proposed as the cause of sensitivity of the basal ganglia to anoxic damage. Certain vascular domains of the basal ganglia are hypoperfused, making it very vulnerable to hypoxia. Hypoxia has been shown to increase striatal extracellular glutamate. The high oxidative metabolism of striatum makes it prone to vascular damage.^7^

The time of onset of dystonia following cerebral anoxia after the perinatal period is variable, ranging from 1 week to 36 months.^8^ We describe a 13-year-old male who developed SD as an acute manifestation of cerebral anoxia following cardiac arrest.

## Case report

**Figure 1:**
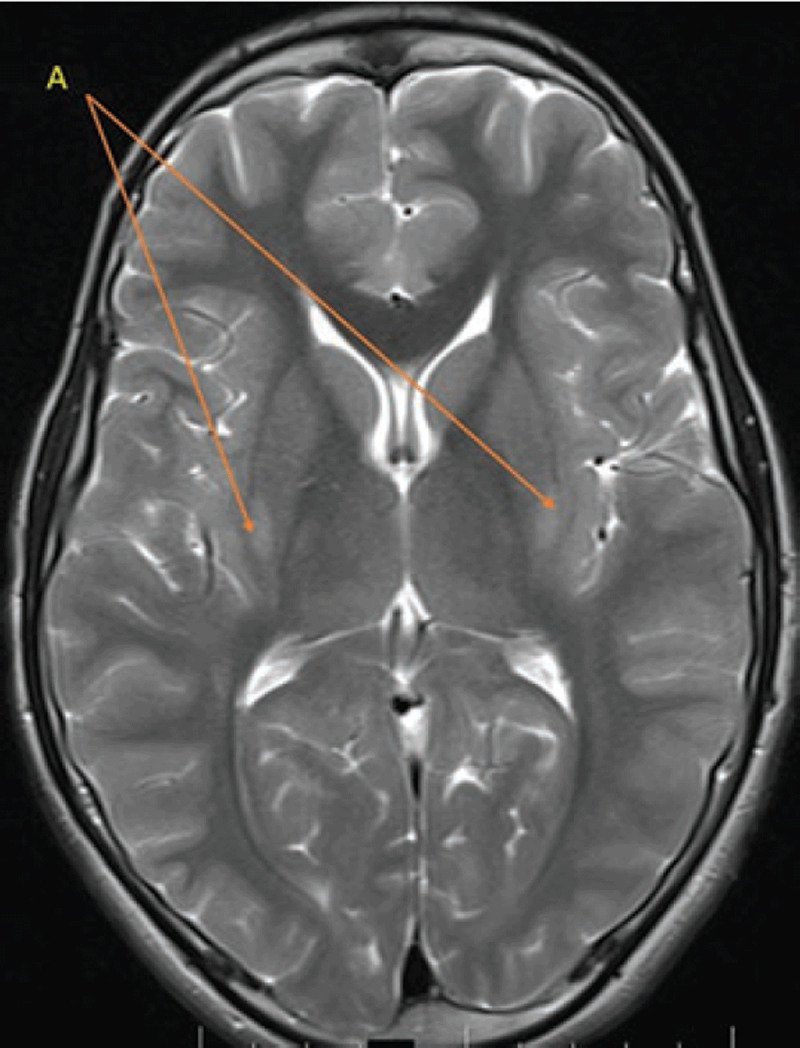
**Selective MRI Image is Displayed with Salient Features.** (A) The axial T2 fluid-attenuated inversion recovery sequence showing hyperintensity in the putamen bilaterally suggesting anoxic injury.

A 13-year-old male with no known comorbidities was well until 20 days before admission to the hospital. While cycling back from school he had developed sudden onset of altered sensorium with loss of consciousness. The patient was given cardiopulmonary resuscitation and revived and subsequently intubated. An echocardiogram showed regional wall motion abnormalities in the anterior wall of the left anterior descending artery, suggesting anterior wall myocardial infarction, and an ejection fraction of 40%. He received aspirin and enoxaparin. Magnetic resonance imaging (MRI) of the brain showed bilateral cortical swelling, fluid-attenuated inversion recovery (FLAIR) and T2 hyperintensities in bilateral thalami, posterior putamen with restriction on diffusion-weighted images suggesting hypoxic-ischemic damage (Figure 1). On the 10th day after the first symptom, the patient developed abnormal posturing of all four limbs with hyperextension of the back (Video 1). On the 16th day, opisthotonic posturing and abnormal posturing of the limbs worsened with no triggering factors. The patient had one episode of tonic-clonic seizure. He had no prior history of chest pain, palpitation, exertional breathlessness, or syncope. There was no family history of dystonia or any other movement disorders. The patient was examined on the 20th day after the first symptom. On examination, the patient was drowsy and did not respond to painful stimuli. His pupils were equally bilaterally reactive. The oculocephalic reflex was present. There was no autonomic dysfunction.

**Video 1:**
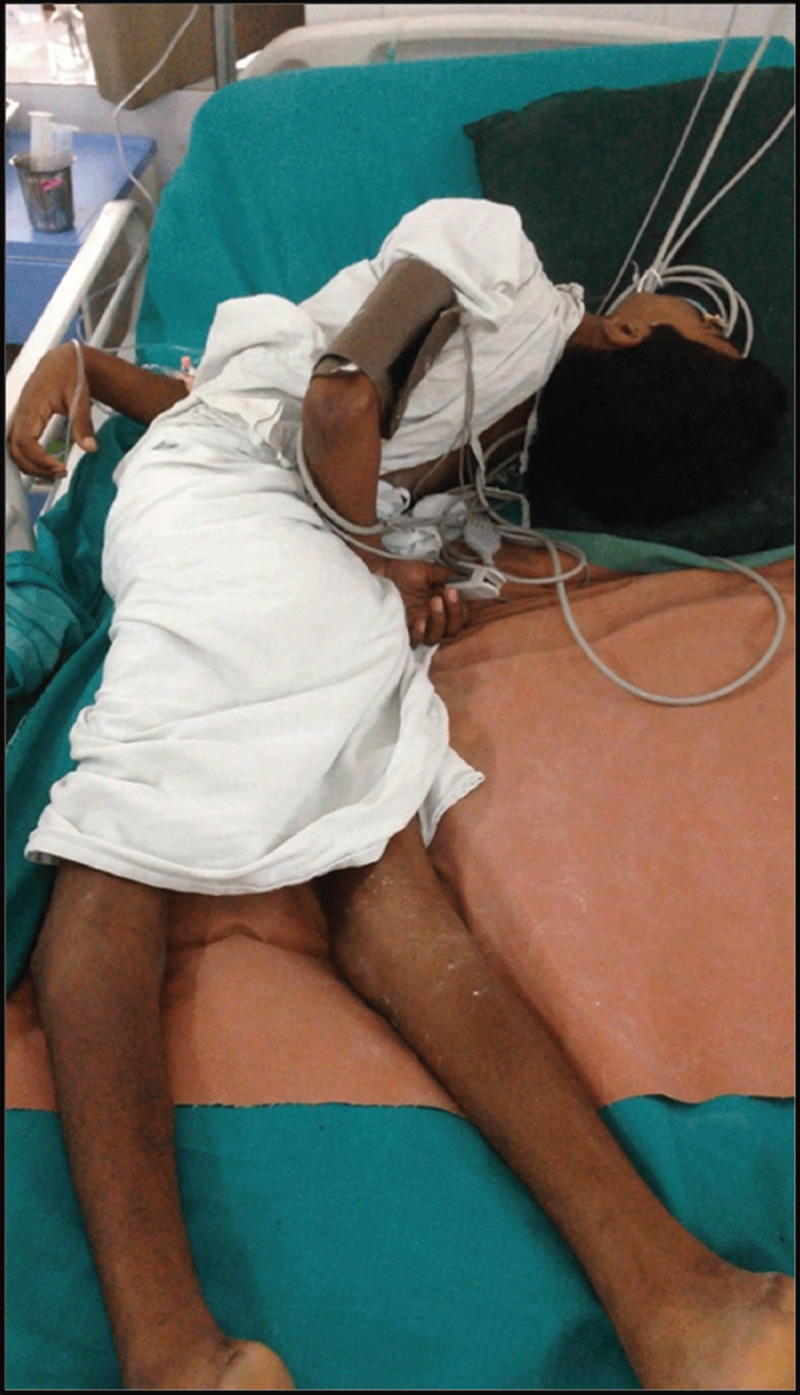
**This Video Grab shows the Opisthotonic Dystonic Posturing in the Patient Following Anoxic Brain Injury.** The patient is seen experiencing opisthotonic posturing and generalized dystonia.

Investigations revealed a creatine phosphokinase (CPK) value of 1,499 IU/L, which progressively increased to 2,411 IU/L in the next 10 days, and his urine was persistently positive for myoglobin. Serum homocysteine was elevated (24.96 μmol/L). Electroencephalography (EEG) suggested diffuse cerebral dysfunction. A repeat echocardiogram showed left-ventricle segmental hypokinesia and the ejection fraction was 47%.

The patient was first started on intravenous promethazine 25 mg twice daily and haloperidol 2.5 mg four times a day. Owing to a lack of improvement, he was subsequently started on tetrabenazine and then trihexylphenidyl. The patient was then intubated and started on diazepam 5 mg per day, increasing to 15 mg per day. There was a significant improvement with tetrabenazine and trihexyphenidyl; the doses were increased to tetrabenazine 75 mg per day and trihexyphenidyl 18 mg per day in divided doses. He was also started on oral baclofen 20 mg per day and levodopa/carbidopa 110 mg three times a day. The patient was started on adequate rehydration and antipyretics for fever and intravenous levetiracetam for seizure. For cardiac dysfunction, dysfunction, he was treated with dual antiplatelet therapy and digoxin 0.25 mg, half a tablet once daily for five days a week. The QT interval was monitored while the patient was on tetrabenazine and there was no prolongation of the QT interval.

The patient had a significant reduction in generalized dystonia by the ninth day of admission to the intensive care unit (ICU), and showed no opisthotonic or dystonic spasms by the 11th day of ICU care. At discharge, the patient was bed bound, his eyes opened spontaneously, all four limbs moved spontaneously, dystonia was less severe, and there was no speech output. The patient was lost to follow-up 6 months after discharge.

## Discussion

Non-progressive cerebral insults such as perinatal hypoxia and kernicterus can cause chorea, athetosis, and dystonia while cerebral infarction has resulted in ballistic choreic or athetoid movements.^9^,^10^ Anoxic brain damage can also cause action myoclonus, tremors, and akinetic rigid syndrome.^14^,^15^,^8^ Involuntary movements may develop at the time of insult or following resolution of acute neurological deficits. In the case of perinatal damage, abnormal movements appear after neurological maturation. Children with perinatal asphyxia showed the earliest movement disorders, at 10-18 months of age.^12^ Dystonia has been associated with lesions of the contralateral putamen, posterior and postero-lateral external globus pallidus, and red nucleus.^11^ In a case series, putamen lesions were found in 14 patients of whom 10 patients had dystonia.^8^ In a review of 12 patients who suffered ischemic insult, six patients developed a pure dystonic syndrome ranging from 1 week to 36 months after the onset of hypoxia that was gradually progressive. Four patients initially developed akinetic- rigid syndrome followed by dystonia.^8^ The age of occurrence of anoxic injury is vital in predicting the outcome, with akinetic-rigid syndrome developing in older patients and dystonia developing in younger patients. The pathophysiological mechanisms underlying age-dependent differences may be because ageing of the nigrostriatal system renders older individuals more susceptible to parkinsonism following anoxia.^8^

SD starting shortly after cerebral anoxia has seldom been reported. Bhatt et al.^8^ reported four patients who developed dystonia ranging from 1-2 weeks after the occurrence of cerebral anoxia, but these patients were not reported to have SD.^8^ Our patient developed dystonia and opisthotonic posturing on the 10th day of cerebral anoxic insult, which worsened on the 16th day and on the 20th day. At the time of examination in the casualty department, he was experiencing continuous dystonic spasms with severe opisthotonus. Pierro et al.^16^ reported a 14-month-old female who suffered cerebral anoxia after nearly drowning. On day 12 she developed decorticate posturing, dystonia, and opisthotonus and torsion spasms and subsequently was transferred to the ICU on day 41 because of SD. She recovered from her dystonic spasms within the next 20 days. Despite the recovery she was still in a vegetative state. Another child was reported, a 15-month male who developed torsion spasms on day 5 after a cerebral anoxic insult. After 3 months, his dystonia progressively worsened culminating in SD requiring an ICU stay of 6 months.^16^ A 19-month-old child was reported to have had developed SD 2 weeks after anoxic brain damage.^25^ Hence, to our knowledge, our patient is the third patient reported in the literature presenting with SD as an acute manifestation of cerebral anoxia.

SD has been described in various conditions such as pantothenate kinase-associated neurodegeneration, perinatal hypoxia, megalencephalic leukoencephathy with subcortical cysts, primary generalized dystonia, Wilson disease, ataxia telangiectasia, and Aristaless related homeobox (ARX) syndrome.^1^,^17^,^18^,^19^,^20^,^21^,^22^,^23^ Fever, infections, exposure to medication, or its abrupt cessation can be triggering factors.^18^ The tonic type of SD has been more commonly seen in males and is associated with higher mortality.^6^

A new definition has been proposed for SD in view of the concurrent occurrence of many hyperkinetic disorders with dystonia.^26^

## Conclusion

SD is rare and life-threatening complication of dystonia in both primary and secondary dystonia and can be a presenting manifestation in patients who have suffered cerebral anoxia. Management of precipitating factors, intensive care, and treatment with multiple antidystonia medications are cardinal to overcome the crisis at the earliest.

## References

[1] Opal P, Tintner R, Jankovic J, Leung J, Breakefield XO, Friedman J, et al. Intrafamilial phenotypic variability of the DYT1 dystonia: from asymptomatic TOR1A gene carrier status to dystonic storm. Mov Disord 2002;17: 339–345. doi: 10.1002/mds.10096 11921121

[2] Dalvi A, Fahn S, Ford B. Intrathecal baclofen in the treatment of dystonic storm. Mov Disord 1998; 13: 611–612. doi: 10.1002/mds.870130344 9613767

[3] Vaamonde J, Narbona J, Weiser R, Garcia MA, Brannan T, Obeso JA. Dystonic storms: a practical management problem. Clin Neuropharmacol 1994; 17: 344–347. doi: 10.1097/00002826-199408000-00006 9316682

[4] Jankovic J, Penn AS. Severe dystonia and myoglobinuria. Neurology 1982; 32: 1195. doi: 10.1212/WNL.32.10.1195 6889706

[5] Marsden CD, Marion MH, Quinn N. The treatment of severe dystonia in children and adults. J Neurol Neurosurg Psychiatry 1984; 47: 1166–1173. doi: 10.1136/jnnp.47.11.1166 6502174 PMC1028082

[6] Fasano A, Ricciardi L, Bentivoglio AR, Canavese C, Zorzi G, Petrovic I, et al. Status dystonicus: predictors of outcome and progression patterns of underlying disease. Mov Disord 2012; 27: 783–788. doi: 10.1002/mds.24981 22488948

[7] Hawker K, Lang AE. Hypoxic ischemic damage of the basal ganglia: case reports and review of the literature. Mov Disord 1990; 5: 219–224. doi: 10.1002/mds.870050306 2388637

[8] Bhatt MH, Obeso JA, Marsden CD. Time course of postanoxic akinetic- rigid and dystonic syndromes. Neurology 1993; 43: 314–317. doi: 10.1212/WNL.43.2.314 8437695

[9] Rose J, Vassar R. Movement disorders due to bilirubin toxicity. Sem Neonatal Fetal Med 2015; 20: 20–25. doi: 10.1016/j.siny.2014.11.002 PMC4388741 25524299

[10] Ghika-Schmid F, Ghika J, Regli F. Hyperkinetic movement disorders after stroke. J Neurol Sci 1997; 152: 109–116. doi: 10.1016/S0022-510X(96)00290-0 9077506

[11] Pettigrew LC, Jankovic J. Hemidystonia :a report of 22 patients and a review of literature. Journal of neurology. Neurosurg Psychiatry 1985; 48: 650–657. doi: 10.1136/jnnp.48.7.650 PMC1028406 4031909

[12] Crothers B, Paine RS. The natural history of cerebral palsy St. Martin’s Griffin; 1988.

[13] Kuoppamäki M, Bhatia KP, Quinn N. Progressive delayed-onset dystonia after cerebral anoxic insult in adults. Mov Disord 2002; 17: 1345–1349. doi: 10.1002/mds.10260 12465080

[14] Fahn S. Posthypoxic action myoclonus: literature review update. Adv Neurol 1986; 43: 157–169. 3080849

[15] Govaerts A, Zandijcke MV, Dehaene I. Posthypoxic midbrain tremor. Mov Disord 1998; 13: 359–361. doi: 10.1002/mds.870130230 9539357

[16] Pierro MM, Bollea L, Di Rosa G, Gisondi A, Cassarino P, GIAnnarelli P, et al. Anoxic brain injury following near-drowning in children. Rehabilitation outcome: Three case reports. Brain Injury 2005; 19: 1147–1155. doi: 10.1080/02699050500149973 16286328

[17] Balas I, Kovacs N, Hollody K. Staged bilateral stereotactic pallidotha lamotomy for life-threatening dystonia in a child with Hallervorden-Spatz disease. Mov Disord 2006; 21: 82–85. doi: 10.1002/mds.20655 16108022

[18] Grosso S, Verrotti A, Messina M, Sacchini M, Balestri P. Management of status dystonicus in children. Cases report and review. Eur J Paediatr Neurol 2012; 16: 390–395. doi: 10.1016/j.ejpn.2011.12.007 22244366

[19] Jankovic J, Penn AS. Severe dystonia and myoglobinuria. Neurology 1982; 32: 1195. doi: 10.1212/WNL.32.10.1195 6889706

[20] Paret G, Tirosh R, Zeev BB, Vardi A, Brandt N, Barzilay Z. Intrathecal baclofen for severe torsion dystonia in a child. Acta Paediatrica 1996; 85: 635–637. doi: 10.1111/j.1651-2227.1996.tb14109.x 8827116

[21] Paliwal VK, Gupta PK, Pradhan S. Gabapentin as a rescue drug in D-penicillamine-induced status dystonicus in patients with Wilson disease. Neurol India 2010; 58: 761. doi: 10.4103/0028-3886.72184 21045506

[22] Ray S, Sidhu RJ, Yadav R, Srinivas D, Pal PK. Refractory status dystonicus in ataxia telangiectasia. Neurol India 2017; 65: 169–172. doi: 10.4103/0028-3886.198206 28084263

[23] Guerrini R, Moro F, Kato M, Barkovich AJ, Shiihara T, McShane MA, et al. Expansion of the first PolyA tract of ARX causes infantile spasms and status dystonicus. Neurology 2007; 69: 427–433. doi: 10.1212/01.wnl.0000266594.16202.c1 17664401

[24] Albanese A. Phenomenology and classification of dystonia: a consensus update. Mov Disord 2013; 28: 863–873. doi: 10.1002/mds.25475 23649720 PMC3729880

[25] Mrkobrada S, Gnanakumar V. The correlation of dystonia severity and serum transaminases in a child with a brain injury. Pediatr Neurol 2014; 51: 573–575. doi: 10.1016/j.pediatrneurol.2014.06.012 25266623

[26] Ruiz-Lopez M, Fasano A. Rethinking status dystonicus. Mov Disord 2017; 32: 1667–1676. doi: 10.1002/mds.27207 29144565

